# Impact of tumour proximity to organs-at-risk in adaptive MR-guided SBRT for central lung tumours and metastases

**DOI:** 10.1016/j.ctro.2025.101079

**Published:** 2025-11-15

**Authors:** Sina Mansoorian, Ala Sami Ismail Salameh, Laura Hamm, Svenja Hering, Diego Kauffmann-Guerrero, Helmut Weingandt, Vanessa da Silva Mendes, Jan Hofmaier, Sebastian Marschner, Nina-Sophie Schmidt-Hegemann, Guillaume Landry, Claus Belka, Stefanie Corradini, Chukwuka Eze

**Affiliations:** a Department of Radiation Oncology, University Hospital, LMU Munich, Munich, Germany; b Department of Medicine V, University Hospital, Munich, Munich, Germany; c Comprehensive Pneumology Center Munich (CPC-M), Member of the German Center for Lung Research (DZL), Munich, Germany; d German Cancer Consortium (DKTK), partner site Munich and German Cancer Research Center (DKFZ), Heidelberg, Germany; e Bavarian Cancer Research Center (BZKF), Munich, Germany

**Keywords:** Adaptive radiotherapy, Central lung tumours, MR-guided radiotherapy, Organ-at-risk sparing, Stereotactic body radiotherapy

## Abstract

**Background:**

Centrally located lung tumours present challenges for SBRT due to elevated toxicity risk. Online adaptive MR-guided radiotherapy (oMRgRT) offers improved target coverage and Organ at risk (OAR) sparing by accounting for interfractional anatomical changes. This study evaluated the dosimetric impact of oMRgRT, with emphasis on tumour location relative to OARs and the clinical benefit of adaptation.

**Methods:**

We retrospectively analysed 36 PTVs across 294 treatment sessions using a 0.35 T MR-Linac. Tumours were categorised by proximity to six critical OARs: proximal bronchial tree (PBT), trachea, heart, great vessels, brachial plexus, and oesophagus. Predicted/reoptimised plans from all fractions were compared to assess improvements in target coverage and OAR sparing. All dosimetric parameters were presented as a percentage of baseline plan metrics. Statistical tests included the Wilcoxon signed-rank test and the Mann-Whitney *U* test.

**Results:**

Adaptive planning significantly improved target volume dosimetry. PTV D_98%_ increased from 92.8 ± 8.7% to 99.9 ± 1% (p < 0.01); V_prescription dose (PD)_ improved from 92.7 ± 5.4% to 97.7 ± 1.1% (p < 0.01). GTV D_98%_ rose from 98.5 ± 5.5% to 100.4 ± 4.3% (p < 0.01), with V_PD_ increasing from 97.7 ± 3.9% to 98.1 ± 3.5% (p < 0.01). Improvements in PTV coverage were observed across all subgroups, with the greatest gains in GTV coverage, most notable in tumours adjacent to the PBT and heart. Tumours near the trachea, great vessels, and brachial plexus showed minimal change. The most significant reductions in OAR doses were also seen in the heart and PBT groups, while proximity to the trachea resulted in minimal benefit**.**

**Conclusion:**

Daily oMRgRT significantly improves target coverage and OAR sparing in centrally located tumours, especially in anatomically complex regions. The adaptive approach enables clinically meaningful trade-offs between tumour coverage and OAR sparing. Further studies are needed to refine adaptation protocols based on tumour sublocation.

## Highlights

• Central lung tumours and metastases pose a treatment challenge due to nearby critical structures and toxicity risk.
• This study analyzed 294 fractions treated with online adaptive MR-guided radiotherapy (oMRgRT)
• Daily plan adaptation improved target coverage and spared organs at risk.
• The greatest benefits were seen in tumours near the proximal bronchial tree and heart.
• Results support oMRgRT for treatment of central lung tumours.

## Introduction

The definition of centrally located lung tumours in the context of stereotactic body radiotherapy (SBRT) varies across groups [1]. However, these definitions consistently emphasise the elevated risk of toxicity when tumours are in close proximity (≤ 2 cm) to critical structures such as the proximal bronchial tree (PBT), trachea, heart, oesophagus, major vessels, brachial plexus, spinal cord, phrenic nerve, and recurrent laryngeal nerve, highlighting the clinical importance of careful dose planning in these high-risk regions [[1], [2], [3], [4]].

Magnetic resonance-guided radiotherapy (MRgRT) has emerged as a relatively novel solution to this problem. The integration of real-time MR imaging with linear accelerators enables superior soft-tissue contrast, precise tumour tracking, and the capability to perform daily online adaptive replanning. This approach allows clinicians to adjust treatment delivery based on daily anatomical variations, thereby improving both target coverage and protection of the organs-at-risk (OARs) [[5], [6], [7], [8], [9], [10]].

In the thoracic region, day-to-day changes in lung inflation, tumour deformation, and variable positions of mediastinal organs can significantly affect dose distribution. Without adaptation, conventional SBRT plans may either underdose the tumour or exceed the constraints for OARs. Online adaptive MRgRT (oMRgRT) has demonstrated feasibility and dosimetric advantages in addressing these issues, particularly in centrally located and anatomically complex tumours [[5], [6], [7], [8], [9], [10], [11], [12], [13]].

Data show that adaptive MRgRT reduces violations of OAR constraints while maintaining or improving target coverage and enhancing OAR protection in thoracic malignancies [5,12].

In addition to the lack of a universally accepted definition of (ultra-)centrally located tumours, there is limited data comparing plan dosimetry across different central tumour locations. The objective of this study was to systematically evaluate the dosimetric impact of daily online MR-guided adaptive radiotherapy in patients with centrally located lung tumours/metastases. We retrospectively compare predicted and reoptimised treatment plans to quantify improvements in planning target volume (PTV) coverage and OAR sparing. This analysis aimed to assess the benefit of adaptation based on tumour location relative to specific OARs.

## Methods

### Patient selection and treatment

This retrospective dosimetric study included 40 consecutive stereotactic treatment series delivered between June 2020 and April 2024 in 36 patients with centrally located lung tumours or metastases, all treated with SBRT prescribed to the 65–80% isodose line. Four cases were excluded to ensure cohort homogeneity, as the minimum dose that covered 95% of the PTV was 95% of the prescription dose (D95%=95%) instead of 100%. Lesions were classified as central if they were within ≤ 2 cm of critical mediastinal structures, such as the PBT, trachea, heart, oesophagus, or major vessels.

### Workflow and delivery of online MR-guided SBRT

The MRIdian platform [14] and the applied workflow have been detailed in earlier reports [10,15,16]. Patients are positioned using dedicated immobilisation devices and typically treated in breath hold to minimise motion. Following an initial MRI simulation and co-registeration of simulation CT for electron density mapping, treatment plans were generated on MRI datasets with Gross Tumour Volume (GTV) to PTV expansion and predefined dose objectives.

Before each fraction, a setup MRI was acquired for patient alignment. The baseline plan is deformably registered to the daily MRI, propagating contours and density information to create a synthetic CT. Predicted dose is calculated on this anatomy. If target coverage is insufficient or OAR constraints are exceeded, an adaptive process is initiated, involving selective re-contouring of OARs near the PTV and plan reoptimisation. Adapted plans are recalculated with consistent parameters and undergo independent Monte Carlo verification before delivery.

For intrafractional tracking, a 2D cine MRI sequence with balanced steady-state free precession (bSSFP) was used. The tracking contour was transferred to the cine slice and expanded to form a gating region of interest (ROI), which enabled online gating of the treatment beam. Patients with respiration-induced target motion were managed using a breath-hold technique.

### Organ-at-risk selection and treatment plan classification

Since dose levels to some OARs were already well below institutional constraints, adaptive planning sometimes allowed dose redistribution − either by increasing dose to lower-risk OARs to improve target coverage or by decreasing dose to higher-risk OARs to stay within constraints. Therefore, unlike other approaches [5], this study focused exclusively on OARs near the tumour that were at risk of receiving clinically significant exposure. Specifically, OARs were included in the analysis if they either received doses exceeding 80% of the institutional dose constraint for the given fractionation or were within 1 cm of the PTV. All constraints were based on institutional protocols and published dose tolerance data [1,[17], [18], [19]].

To assess the impact of anatomical proximity on adaptation results, tumours were grouped by the nearest OAR into six categories: PBT, oesophagus, trachea, heart, great vessels, and brachial plexus. Treatment plans were also examined based on the use of planning risk volumes (PRVs), evaluating their influence on PTV coverage and OAR dose. Plan distribution by group was as follows: PBT (13 patients, 125 paired plans, 1 session without adaptation), trachea (2 patients, 20 paired plans), heart (11 patients, 72 paired plans, 2 sessions without adaptation), great vessels (6 patients, 42 paired plans, 1 session without adaptation), and brachial plexus (4 patients, 35 paired plans). No patients were classified in the oesophagus group. Each session included three plan types: baseline (post-simulation), predicted (n = 298), and reoptimised (n = 294) using daily oMRgRT. All plans were created and evaluated on a 0.35 T MR-Linac system.

### Dosimetric evaluation and metrics

Dose-volume Parameters for PTV, GTV, and OARs from predicted and reoptimised plans were presented as a percentage of baseline plan metrics to enable comparison across different fractionation regimens, except for V_prescription dose (PD)_, which was reported as its original percentage value. Key metrics included V_PD_ - the proportion of the target volume receiving at least the prescription dose - and D_xx%_ or D_xycc_, representing the minimum dose received by xx% or xy cc of a given structure, to assess improvements in target coverage and OAR sparing.

### Statistical analysis

Statistical analysis was performed using the Wilcoxon signed-rank test for paired data and the Mann–Whitney *U* test for unpaired data that did not follow a normal distribution, as assessed by the Shapiro–Wilk test. A two-tailed p-value <0.05 was considered statistically significant. Data processing and statistical analyses were performed using Microsoft Excel 365 (Microsoft Corp.), SPSS Statistics version 29 (IBM Corp.), and GraphPad Prism version 10.5.0 (for Windows; GraphPad Software, Boston, Massachusetts, USA).

## Results

Table 1 and Supplementary Table 1 summarise the patient and treatment characteristics of the study cohort. Three patients were treated with two GTVs in a single planning volume. Four patients underwent two courses of radiation. Overall, most treatment sessions showed measurable improvements in both target coverage and OAR sparing with adaptive radiotherapy. PTV V_PD_ improved in 87.4% of sessions, while PTV D_98%_ improved in 86.4% of sessions. Dose reductions to OARs, assessed by D_0.03cc_, were also frequently achieved: improvements were observed in 71.7% of sessions for the proximal bronchial tree, 66.7% for the trachea, 65.6% for the heart, 63.6% for the great vessels, 60% for the brachial plexus, 60% for the spinal cord, and 56.6% for the oesophagus.

**Table 1:** Patient and treatment characteristics.

Patient and tumour characteristics	Total
Total Patients, n (%)	32 (100%)
Sex	
Female, n (%)	19 (63.9%)
Male, n (%)	13 (36.1%)
Age [years], median (range)	63.0 (40.0–84.0)
Total PTVs, n (%)	36 (100%)
Total GTVs, n (%)	39 (100%)
Plans with 2 PTVs, n (%)	3 (7.7%)
Treatment fractions, n	
Total fractions, n (%)	298 (100%)
Treatment sessions with adapted plan, n (%)	294 (98.7%)
Treatment sessions with predicted plan, n (%)	4 (1.3%)
Prescribed dose	
5 to 50 Gy (10 Fractions) 80% IDL, n (%)	23 (63.9%)
10 to 50 Gy (5 Fractions) 80% IDL, n (%)	7 (19.4%)
7.5 to 60 Gy (8 Fractions) 80% IDL, n (%)	3 (8.3%)
15 to 45 Gy (3 Fractions) 65% IDL, n (%)	2 (5.6%)
13.5 to 40.5 Gy (3 Fractions) 65% IDL, n (%)	1 (2.8%)
Target volumes	
GTV [cc], median (interquartile range)	6.4 (3.0–13.6)
PTV [cc], median (interquartile range)	17.1 (9.5–32.6)
GTV to PTV margin	
5 mm, n (%)	34 (87.2%)
3 mm, n (%)	5 (12.8%)
GTV without contact with OAR, n (%)	23 (59.0%)
GTV in contact with OAR, n (%)	16 (41.0%)
PBT, n (%)	4 (10.3%)
Trachea, n (%)	1 (2.6%)
Heart, n (%)	3 (7.7%)
Great vessels, n (%)	3 (7.7%)
Brachial plexus, n (%)	2 (5.1%)
Oesophagus, Trachea and Great vessels, n (%)	1 (2.6%)
PBT, Oesophagus, Trachea and Great vessels, n (%)	1 (2.6%)
PBT, Trachea, Heart and Great vessels, n (%)	1 (2.6%)
PRV for OAR, total, n (%)	28 (77.8%)
0 mm, n (%)	13 (36.1%)
2 mm, n (%)	2 (5.6%)
3 mm, n (%)	13 (36.1%)

Abbreviations: GTV – Gross Tumour Volume; Gy – Gray; IDL – Isodose Line; n – Number; OAR – Organ-at-Risk; PBT – Proximal Bronchial Tree; PRV – Planning Risk Volume; PTV – Planning Target Volume.

Tumours located near the PBT and trachea were consistently treated using 8–10 fraction regimens. In two such cases (Cases 9 and 13, please refer to Supplementary Table 1), where tumours were adjacent to the PBT, a dose of 7.5 Gy per fraction was delivered to a total dose of 60 Gy, prescribed to the 80% isodose line, resulting in a BED_10_ of 105 Gy. In both cases, the PTV did not directly abut any critical OARs; the closest structure was the lobar bronchus, located 1–2 cm from the target. Additionally, in one case, the GTV-to-PTV expansion was only 3 mm, and no direct contact with OARs was observed.

Adaptation significantly improved target volume dosimetry across all key parameters. For the PTV, V_PD_ increased from a mean of 92.7 ± 5.4% in predicted plans to 97.7 ± 1.1% in reoptimised plans (n = 294 paired comparisons; two-tailed Wilcoxon signed-rank test, p < 0.01). For the GTV, V_PD_ also improved significantly, although to a lesser extent, from 97.7 ± 3.9% to 98.1 ± 3.5% (p < 0.01). Full results are provided in Table 2A, and a schematic illustration of the dosimetric differences is shown in Fig. 1A as a spider plot.

**Table 2A:** Comparison of predicted and reoptimised plans for target volumes.

Dose-Volume parameter as % of baseline plan n (paired) = 294		Predicted	Reoptimised		Predicted vs. Reoptimised
		Mean ± SD	Mean ± SD	p-value (Wilcoxon test)	Δ Median (95%CI)
PTV	D_98%_	92.8 ± 8.7%	99.9 ± 1%	<0.01	4.7 (6.1 to 8)
	D_95%_	95.6 ± 5.4%	100.0 ± 0.7%	<0.01	2.9 (3.8 to 5)
	D_50%_	99.7 ± 1.8%	100.0 ± 1%	<0.01	0.4 (0.1 to 0.6)
	D_mean_	99.2 ± 2%	99.9 ± 1.3%	<0.01	0.6 (0.5 to 1)
	D_2%_	101.2 ± 3.5%	100.2 ± 2.6%	<0.01	−0.7 (−1.3 to-0.8)
GTV	D_98%_	98.5 ± 5.5%	100.4 ± 4.3%	<0.01	1.4 (1.2 to 2.5)
	D_95%_	98.9 ± 3.9%	100.1 ± 3.1%	<0.01	0.9 (0.7 to 1.7)
	D_50%_	99.9 ± 2.1%	100 ± 1.3%	0.3	0.1 (−0.2 to 0.4)
	D_mean_	99.8 ± 2%	99.9 ± 1.4%	0.4	0.1 (−0.2 to 0.4)
	D_2%_	100.9 ± 2.5%	99.8 ± 1.1%	<0.01	−0.8 (−1.4 to −0.8)

**Fig. 1A:**
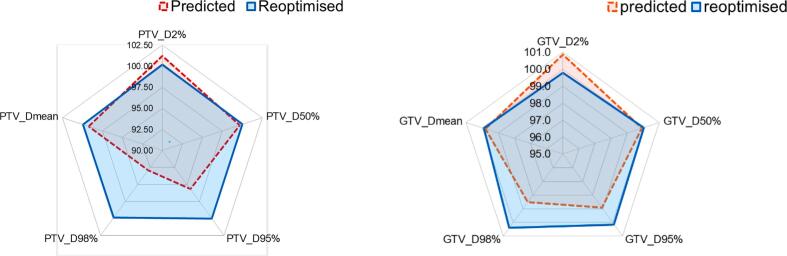
Comparison of PTV and GTV dose-volume parameters between non-adapted (red) and adapted (blue) plans. Mean values for D_98%_, D_95%_, D_50%_, D_2%_, and D_mean_ are presented. Adaptive planning resulted in improved target coverage, as indicated by increased D_98%_, D_95%_, D_50%_, and D_mean_ values for both PTV and GTV, while avoiding increases in the high-dose region represented by D_2%_. (For interpretation of the references to colour in this figure legend, the reader is referred to the web version of this article.)

Adaptive planning also led to consistent reductions in maximum point doses (D_0.03cc_) across multiple organs-at-risk. The most substantial reductions were observed in the PBT (−5%), heart (−4.8%), and brachial plexus (−4.7%). All reductions were statistically significant (p < 0.05). Full results are provided in Table 2B and Fig. 1B.

**Table 2B:** Overall comparison of predicted versus reoptimised OAR parameters, regardless of nearest critical organ-at-risk (OAR).

Dose-Volume parameter as a % of baseline plan	N (paired)	Predicted	Reoptimised	p-value (Wilcoxon test)	Predicted vs. Reoptimised
		Mean ± SD	Mean ± SD		Δ Median (95%-CI)
Proximal bronchial tree, D_0.03cc_	123	103.7 ± 14.0%	98.0 ± 12.9%	<0.01	−5.0 (−7.9 to −3.4)
Oesophagus, D_0.03cc_	120	105.5 ± 15.8%	100 ± 7.6%	<0.01	−2.6 (−8.5 to −2.6)
Trachea*, D_0.03cc_	45	102.9 ± 7.5%	98.4 ± 4.9%	<0.01	−4.0 (−7.0 to −2.0)
Heart, D_0.03cc_	137	102.9 ± 18.5%	97.4 ± 14.7%	<0.01	−4.8 (−8.1 to −2.9)
Great vessels, D_0.03cc_	162	100.2 ± 8.6%	98 ± 7.6%	<0.01	−1.9 (−3.5 to −0.9)
Brachial plexus, D_0.03cc_	35	102.1 ± 18.7%	96.4 ± 10.7%	0.04	−4.7 (−10.9 to −0.4)
Spinal cord, D_0.03cc_	39	105.5 ± 15.8%	100 ± 7.6%	0.04	−3.7 (−9.7 to −0.8)

* It should be noted that, in the pooled analysis of all cases, tracheal doses differed significantly between adaptive and predicted plans. This difference was likely driven by tumours located centrally near the proximal bronchial tree or the heart. However, as shown in Table C, the difference for the trachea was no longer statistically significant when tumours of the superior mediastinum adjacent to the trachea were analysed separately.

**Fig. 1B:**
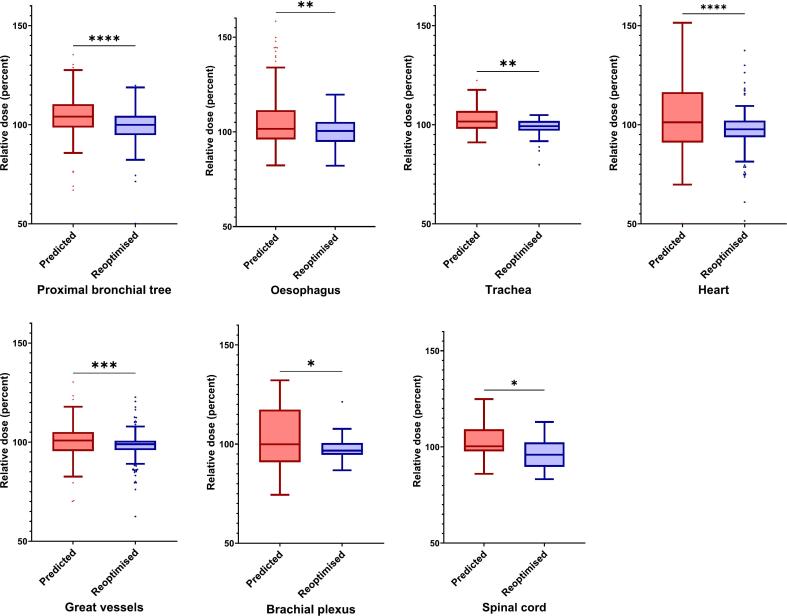
Comparison of D_0.03cc_ doses to organs at risk (OARs) across all tumour locations. Included OARs either received more than 80% of their institutional dose constraint or were located within 1  cm of the planning target volume (PTV). Values are shown for predicted (left, red) and reoptimised (right, blue) plans, relative to the baseline plans. Boxes indicate the interquartile range (IQR), with the horizontal line representing the median. Whiskers extend to 1.5 × IQR. *p < 0.05, **p < 0.001, ***p < 0.0001, ****p < 0.00001. (For interpretation of the references to colour in this figure legend, the reader is referred to the web version of this article.)

The comparison of plans based on tumour location, stratified by the nearest OAR defining centrality (≤2 cm proximity), also showed a significant improvement in PTV dose metrics across all tumour locations. However, improvements in GTV dose metrics through reoptimisation were only statistically significant for tumours located near the PBT and heart. Additionally, reductions in OAR D0.03 cc were statistically significant for all locations except for tumours adjacent to the trachea (Table 2C).

**Table 2C:** Comparison of predicted and reoptimised plans stratified by the nearest OAR defining centrality (≤2 cm proximity).

Metric	Proximal bronchial tree	Trachea	Heart	Great vessels	Brachial plexus
PTV V_PD_					
Δ Median	3.7	5.6	4.2	3.3	1.6
95%-CI	−0.8 to 16.5	−0.6 to 10.9	−1.5 to 18	−0.3 to 19.6	−2.4 to 7.1
p-value	<0.01	<0.01	<0.01	<0.01	<0.01
PTV D_98%_					
Δ Median	5.2	5	4.5	4.1	1.9
95%-CI	−1.3 to 28.3	−0.8 to 9.6	−3.3 to 24.4	−0.4 to 19.1	−2.4 to 21.5
p-value	<0.01	<0.01	<0.01	<0.01	<0.01
GTV V_PD_					
Δ Median	0.0	1.1	0.00	0.00	0.00
95%-CI	−2.2 to 4.5	−9.2 to 6.3	−2.7 to 7.3	−0.6 to 8	−2.0 to 4
p-value	0.01	0.09	<0.01	0.4	0.1
GTV D_98%_					
Δ Median	1.1	1.7	2.6	0.82	0.62
95%-CI	−8.4 to 14.2	−12.7 to 12.4	−9.9 to 20	−3.5 to 8	−9 to 13.4
p-value	<0.01	0.3	<0.01	0.09	0.3
OAR D_0.03cc_					
Δ Median	−6.1	−1.8	−7.6	−1.9	−4.7
95%-CI	−21.6 to 26.4	−17.3 to 9.7	–33.0 to 11.1	–23.1 to 11.9	−37.5 to 23.4
p-value	<0.01	0.2	<0.01	0.01	0.04

Legend: Difference between predicted and reoptimised values (Predicted – Reoptimised).

Range — Minimum to maximum across patients, OAR − Organ-at-Risk.

**Fig. 2:**
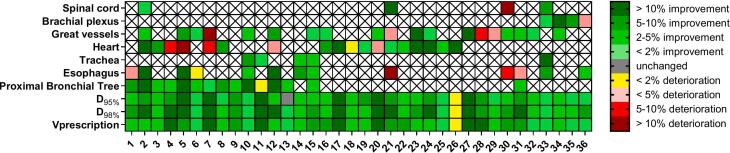
PTV coverage and OAR dose changes across 36 treatment plans. This categorical heatmap visualises changes in PTV coverage (V_prescription_, D_98%_ and D_95%_) and organ-at-risk (OAR) sparing for structures located within 1 cm of the gross tumour volume (GTV) across 36 adaptive treatment series. Color coding illustrates the magnitude and direction of change: green indicates improvement in either PTV coverage or OAR protection, yellow to dark red denote deterioration − such as reduced PTV coverage or increased OAR dose − and grey represents no significant change. To enhance interpretability, the degree of change was stratified into the following thresholds: <2%, 2% to <5%, 5% to 10%, and >10%.

To better illustrate patient-specific trade-offs between target coverage and OAR sparing, a categorical heatmap was used to depict dosimetric changes across all treatment plans (Fig. 2). Each patient’s adaptation outcome is presented relative to the nearest OARs. Notably, only one case (plan number 26) demonstrated a deliberate reduction in target coverage to achieve a >10% improvement in cardiac sparing. This decision was made for a patient with a tumour adjacent to the heart and a history of prior thoracic irradiation, where cumulative cardiac dose was a critical consideration. In all other cases, observed reductions in target or OAR doses remained below clinically relevant constraint thresholds and were deemed acceptable.

In five cases, GTV was expanded by 5 mm to create PTV, with no significant decline in GTV coverage (p > 0.05).

## Discussion

Radiotherapy for centrally located lung tumours remains a significant clinical challenge due to the elevated risk of toxicity [20,21]. Our study demonstrates that online stereotactic MR-guided adaptive radiotherapy significantly improves dosimetric outcome for centrally located lung tumours and metastases. Daily plan adaptation resulted in improved target coverage for both the PTV and GTV compared to non-adaptive plans, while simultaneously reducing the dose to surrounding OARs. These findings align with prior studies that have demonstrated improved PTV coverage and greater sparing of OAR through the use of MR-guided SBRT [5,6,13].

In our study, significant improvements in PTV dose metrics were observed across all subgroups. However, meaningful gains in GTV coverage were primarily observed in tumours adjacent to the PBT and heart, which are regions characterised by greater anatomical mobility and are therefore more prone to pronounced interfractional variation [22]. In contrast, tumours near the trachea, great vessels, and brachial plexus exhibited minimal changes in GTV coverage, with no statistically significant benefit observed from adaptation. The challenges associated with tumours adjacent to the PBT were examined in detail in quality assurance (QA) reports from two ongoing randomised trials. In the VALOR trial, a phase 3 randomised study comparing surgery to SBRT in operable early-stage non-small cell lung cancer, the authors reported accurate contouring of the PBT in 75% of cases [23]. Additionally, the design and pre-trial QA report for the Nordic STRICTLUNG and STARLUNG trials, investigating SBRT for central and ultra-central lung tumours, respectively, demonstrated that even small variations in OAR delineation could result in substantial OAR overdosing. Both studies highlight the nuances and the importance of having extensive QA procedures in place before initiating SBRT trials [24].

Upper mediastinal tumours close to the trachea demonstrated stable dosimetry between predicted and adapted plans, reflecting limited respiratory motion in that region. Similarly, although dose to the brachial plexus was reduced in some cases, adaptive planning had no significant effect on target coverage. These findings are consistent with data from La Rosa et al., who reported that tolerance dose exceedance occurred in 57.4% of PBT cases, but only in 14.4% and 10.3% of trachea and brachial plexus cases, respectively [12].

Regarding OAR sparing, the most significant dose reductions were observed in tumours near the heart, followed by the PBT, great vessels, and brachial plexus. The trachea group again showed minimal dosimetric change, likely due to its relatively stable anatomical position and decreased sensitivity to respiratory shifts. These results indicate that the benefit of adaptive planning is most evident in dynamic anatomical regions such as the hilum and pericardial space, where tumour-OAR relationships vary more considerably from day to day. However, as reported in the recently published guidelines from the American Radium Society (ARS), in their recommendations for different scenarios for (ultra-)central locations, structures that seemed most concerning for the expert panel included PBT (which includes the inferior 2 cm of the trachea), oesophagus and upper/superior trachea, which the panel agreed should be treated with equipoise to the PBT [4].

The prescribed regimens corresponded to a median single-fraction dose of 5 Gy (range 5–15 Gy) and a median total dose of 50 Gy (range 40.5–60 Gy), with prescriptions defined at a median of 80% IDL (range 65–80%). The most frequently used regimen was 10 fractions of 5 Gy (total 50 Gy at the 80% isodose line), representing 63.9% of all treatment courses (23/36). This prescription corresponded to a D_max_ of 62.5 Gy within the target volume. Given the moderate prescription levels, adherence to OAR dose constraints was generally well maintained [1,17]. Puckett et al. propose a D_0.035cc_ constraint of 62.9 Gy for the great vessels [17], slightly higher than the expected D_max_ within the GTV in our protocol. As a result, constraint violations for the great vessels were infrequent when treating with this 10-fraction regimen. Similarly, published dose limits for the trachea and main bronchus, such as a D_0.035cc_ threshold of 59 Gy (118% of a 50 Gy prescription), were generally achievable when high-dose heterogeneity was minimised and PRVs were incorporated. However, the primary limitation of this fractionation scheme lies in its relatively low biologically effective dose (BED_10_), which is approximately 75 Gy, well below the ∼100 Gy BED typically considered necessary for ablative SBRT [25,26].

Several studies have explored higher dose regimens for central and ultra-central tumours, reporting varying toxicity outcomes [[27], [28], [29]]. The Nordic HILUS study administered a total dose of 56 Gy delivered in 8 fractions of 7 Gy each, prescribed to the 67% IDL (∼150% of the PD), reporting G3+ toxicity in 34% of cases [27]. In two previous Dutch retrospective analyses using a fractionation scheme of 5 Gy × 12 (BED_10_ = 90 Gy) with varying definitions of ultra-central tumours, a D_max_ less than 140–145% of the prescription dose was associated with G3+ toxicity in 38% and 21% of cases, respectively [30,31]. Additionally, the recent ARS guidelines, along with the Radiosurgery Society and International Stereotactic Radiosurgery Society, all recommend among other regimens 60–70 Gy in 8–10 fractions for ultra-central tumours, as well as 50–60 Gy in 5 fractions or 60–70 Gy in 8–10 fractions for central tumours [4,32,33]. Currently, a German phase 1 dose escalation study (MAGELLAN) is investigating SBRT for ultra-central lung tumours with a starting dose level of 10 × 5.5 Gy and 0.5 Gy per fraction dose increments up to 6.5 Gy [34]. The potential for dose escalation remains a key therapeutic goal, especially for ultra-central tumours where safely delivering an effective dose to achieve tumour control is most challenging [34,35]. In this context, stereotactic MR-guided adaptive radiotherapy provides a promising strategy by allowing precise dose modulation and adaptive sparing of nearby OARs, thereby effectively expanding the therapeutic window in these high-risk cases. However, the validation of these dosimetric improvements with adaptations and MRgRT clinical trials is essential to determine whether they result in tangible benefits such as reduced toxicity, improved tumour control, or prolonged overall survival.

In five of our patients, the GTV was expanded to the PTV using a minimal isotropic margin of 3  mm, enabled by the use of online gating. This approach could significantly reduce radiation exposure to adjacent OARs and improve planning flexibility. However, data show that smaller volumetric expansion of GTV to PTV is associated with a higher risk of death in centrally located lung tumours [36]. Further, the true clinical significance of such narrow margins remains uncertain and requires further investigation. The use of PRV margins depends on clinical judgment and is usually individualised based on tumour location and proximity to critical structures. Evidence from secondary analyses of the SABR-5 trial suggests that small reductions in target coverage, when made to respect PRV-defined constraints, do not necessarily compromise local control [37].

## Limitations

The primary limitation of this study is the predominant use of moderate-dose fractionation (e.g., 5 Gy × 10), which results in a lower biologically effective dose and may restrict how well our findings apply to high-dose, ablative regimens used in ultra-central tumours. However, due to discrepancies reported in high-grade toxicity, this might be viewed as a safer approach. A second major limitation is the heterogeneity in treatment regimens and use of PRVs; patients received different doses and fractionation schedules delivered to the PTV or PTV optimisation structure, which may reflect varying preferences by treating physicians and could confound comparisons and weaken subgroup-specific conclusions. In the next stage, we are preparing a manuscript on the clinical outcomes of our patients treated with this regimen and plan to homogenise dose prescription and planning techniques. Nevertheless, in the ARS guidelines, planning techniques such as decreasing PTV margins, applying dose constraints to PRVs, contouring critical OARs on four-dimensional scans, and limiting hotspots within the internal target volume to 120% of the prescription dose are generally considered appropriate depending on the situation [4]. These recommendations are universal and likely address non-adaptive, CT-based treatment delivery. Given the inherent advantages of oMRgRT, less conservative approaches may potentially be applied. A third significant limitation is the retrospective design. Additional limitations include small sample sizes in certain subgroups − such as tumours near the trachea and brachial plexus − that restrict statistical power and the applicability of results to these specific anatomical locations.

## Conclusions

Daily online adaptive oMRgRT significantly improves PTV coverage and reduces OAR doses in centrally located lung lesions, with the most pronounced benefits observed in tumours near mobile structures such as the PBT and heart. Adaptive planning enables tailored trade-offs between target volume coverage and OAR sparing, potentially expanding the therapeutic window, particularly for ultra-central lung tumours.

Subgroup analysis showed that defining central tumours solely by proximity to OARs does not consistently capture dosimetric complexity or predict the degree of benefit from adaptive MR-guided SBRT. Tumour location influenced both the choice of fractionation regimen and the degree of planning complexity, reflecting anatomical and clinical factors.

These findings support the routine use of oMRgRT in complex and high-risk thoracic cases. However, prospective studies are needed to confirm whether dosimetric improvements translate into clinical benefits and to refine adaptation protocols across varying tumour locations.

## Disclosures

The authors declare the following financial interests/personal relationships which may be considered as potential competing interests: The Department of Radiation Oncology of the LMU University Hospital, LMU Munich has research agreements with Elekta, Brainlab, and C-RAD. Claus Belka reports receiving grants or contracts from ViewRay, Brainlab, and Elekta; payment or honoraria from Bristol-Myers Squibb, Roche, Merck, AstraZeneca, Opasca, C-RAD, Elekta, and ViewRay; receiving support for attending meetings or travel from Bristol-Myers Squibb, Roche, Merck, AstraZeneca, Elekta, and ViewRay; and having a leadership or fiduciary role with ESTRO, all outside the submitted work. Stefanie Corradini reports research grants and speaker fees/travel support from Elekta, ViewRay and Brainlab. Chukwuka Eze reports consulting fees from AstraZeneca, payment or honoraria from AstraZeneca outside the submitted work and received funding in form of a research grant from the German Cancer Aid outside the submitted work.

## Statements and declarations

No funding was received for conducting this study. The authors have no competing interests to declare that are relevant to the content of this article.

## CRediT authorship contribution statement

**Sina Mansoorian:** Data curation, Formal analysis, Methodology, Validation, Visualization, Writing – original draft, Writing – review & editing. **Ala Sami Ismail Salameh:** Data curation, Formal analysis, Methodology, Validation, Visualization, Writing – original draft, Writing – review & editing. **Laura Hamm:** Data curation, Formal analysis, Methodology, Validation, Visualization, Writing – review & editing. **Svenja Hering:** Data curation, Formal analysis, Methodology, Validation, Visualization, Writing – review & editing. **Diego Kauffmann-Guerrero:** Data curation, Formal analysis, Methodology, Validation, Visualization, Writing – review & editing. **Helmut Weingandt:** Formal analysis, Methodology, Validation, Visualization, Writing – review & editing. **Vanessa da Silva Mendes:** Formal analysis, Methodology, Validation, Visualization, Writing – review & editing. **Jan Hofmaier:** Formal analysis, Methodology, Validation, Visualization, Writing – review & editing. **Sebastian Marschner:** Data curation, Formal analysis, Methodology, Validation, Visualization, Writing – review & editing. **Nina-Sophie Schmidt-Hegemann:** Conceptualization, Formal analysis, Methodology, Validation, Visualization, Writing – review & editing. **Guillaume Landry:** Conceptualization, Formal analysis, Methodology, Validation, Writing – review & editing. **Claus Belka:** Conceptualization, Formal analysis, Methodology, Validation, Writing – review & editing, Funding acquisition, Investigation, Project administration, Supervision. **Stefanie Corradini:** Conceptualization, Formal analysis, Methodology, Validation, Writing – review & editing, Funding acquisition, Investigation, Project administration, Supervision. **Chukwuka Eze:** Conceptualization, Data curation, Formal analysis, Funding acquisition, Methodology, Supervision, Visualization, Writing – original draft, Writing – review & editing.

## Declaration of competing interest

The authors declare that they have no known competing financial interests or personal relationships that could have appeared to influence the work reported in this paper.

## References

[1] Chang J.Y., Bezjak A., Mornex F., Committee I.A.R.T. (2015). Stereotactic ablative radiotherapy for centrally located early stage non-small-cell lung cancer: what we have learned. J. Thorac. Oncol..

[2] Timmerman R., McGarry R., Yiannoutsos C., Papiez L., Tudor K., DeLuca J. (2006). Excessive toxicity when treating central tumors in a phase II study of stereotactic body radiation therapy for medically inoperable early-stage lung cancer. J. Clin. Oncol..

[3] Adebahr S., Collette S., Shash E., Lambrecht M., Le Pechoux C., Faivre-Finn C. (2015). LungTech, an EORTC Phase II trial of stereotactic body radiotherapy for centrally located lung tumours: a clinical perspective. Br. J. Radiol..

[4] Park H.S., Rimner A., Amini A., Chang J.Y., Chun S.G., Donington J. (2024). Appropriate use criteria (AUC) for the management of non-small cell lung cancer in a central/ultra-central location: guidelines from the American radium society. J. Thorac. Oncol..

[5] Finazzi T., Palacios M.A., Spoelstra F.O.B., Haasbeek C.J.A., Bruynzeel A.M.E., Slotman B.J. (2019). Role of on-table plan adaptation in MR-guided ablative radiation therapy for central lung tumors. Int. J. Radiat. Oncol. Biol. Phys..

[6] Henke L.E., Olsen J.R., Contreras J.A., Curcuru A., DeWees T.A., Green O.L. (2019). Stereotactic MR-guided online adaptive radiation therapy (SMART) for ultracentral thorax malignancies: results of a phase 1 trial. Adv. Radiat. Oncol..

[7] Regnery S., Katsigiannopulos E., Hoegen P., Weykamp F., Sandrini E., Held T. (2023). To fly or not to fly: Stereotactic MR-guided adaptive radiotherapy effectively treats ultracentral lung tumors with favorable long-term outcomes. Lung Cancer.

[8] Crockett C.B., Samson P., Chuter R., Dubec M., Faivre-Finn C., Green O.L. (2021). Initial clinical experience of MR-guided radiotherapy for non-small cell lung cancer. Front. Oncol..

[9] Nierer L., Eze C., da Silva M.V., Braun J., Thum P., von Bestenbostel R. (2022). Dosimetric benefit of MR-guided online adaptive radiotherapy in different tumor entities: liver, lung, abdominal lymph nodes, pancreas and prostate. Radiat. Oncol..

[10] Hering S., Nieto A., Marschner S., Hofmaier J., Schmidt-Hegemann N.-S., da Silva M.V. (2024). The role of online MR-guided multi-fraction stereotactic ablative radiotherapy in lung tumours. Clin. Transl. Radiat. Oncol..

[11] Merckel L.G., Pomp J., Hackett S.L., van Lier A., van den Dobbelsteen M., Rasing M.J.A. (2024). Stereotactic body radiotherapy of central lung tumours using a 1.5 T MR-linac: First clinical experiences. Clin Transl. Radiat. Oncol..

[12] La Rosa A., Mittauer K.E., Bassiri N., Rzepczynski A.E., Chuong M.D., Yarlagadda S. (2024). Accelerated hypofractionated magnetic resonance guided adaptive radiation therapy for ultracentral lung tumors. Tomography..

[13] Bryant J.M., Sim A.J., Feygelman V., Latifi K., Rosenberg S.A. (2023). Adaptive hypofractionted and stereotactic body radiotherapy for lung tumors with real-time MRI guidance. Front. Oncol..

[14] Klüter S. (2019). Technical design and concept of a 0.35 T MR-Linac. Clin Transl. Radiat. Oncol..

[15] Mutic S., Dempsey J.F. (2014). The ViewRay System: magnetic resonance–guided and controlled radiotherapy. Semin. Radiat. Oncol..

[16] Eze C., Lombardo E., Nierer L., Xiong Y., Niyazi M., Belka C. (2022). MR-guided radiotherapy in node-positive non-small cell lung cancer and severely limited pulmonary reserve: a report proposing a new clinical pathway for the management of high-risk patients. Radiat. Oncol..

[17] Puckett L.L., Titi M., Kujundzic K., Dawes S.L., Gore E.M., Katsoulakis E. (2023). Consensus quality measures and dose constraints for lung cancer from the veterans affairs radiation oncology quality surveillance program and ASTRO expert panel. Pract. Radiat. Oncol..

[18] Timmerman R. (2022). A story of hypofractionation and the table on the wall. Int. J. Radiat. Oncol. Biol. Phys..

[19] Gerhard S.G., Palma D.A., Arifin A.J., Louie A.V., Li G.J., Al-Shafa F. (2021). Organ at risk dose constraints in SABR: a systematic review of active clinical trials. Pract. Radiat. Oncol..

[20] Manyam B.V., Verdecchia K., Videtic G.M.M., Zhuang T., Woody N.M., Wei W. (2020). Validation of RTOG 0813 proximal bronchial tree constraints for pulmonary toxicity with stereotactic body radiation therapy for central non-small cell lung cancer. Int. J. Radiat. Oncol. Biol. Phys..

[21] Milano M.T., Chen Y., Katz A.W., Philip A., Schell M.C., Okunieff P. (2009). Central thoracic lesions treated with hypofractionated stereotactic body radiotherapy. Radiother. Oncol..

[22] Vasquez Osorio E.M., McCallum H., Bedair A., Faivre-Finn C., Haughey A., van Herk M. (2020). Protecting the heart: a practical approach to account for the full extent of heart motion in radiation therapy planning. Int. J. Radiat. Oncol. Biol. Phys..

[23] Ritter T.A., Timmerman R.D., Hanfi H.I., Shi H., Leiner M.K., Feng H. (2025). Centralized quality assurance of stereotactic body radiation therapy for the veterans affairs cooperative studies program study number 2005: A phase 3 randomized trial of lung cancer surgery or stereotactic radiotherapy for operable early-stage non-small cell lung cancer (VALOR). Pract. Radiat. Oncol..

[24] Hoffmann L., Persson G., Nygård L., Nielsen T., Borrisova S., Gaard-Petersen F. (2022). Thorough design and pre-trial quality assurance (QA) decrease dosimetric impact of delineation and dose planning variability in the STRICTLUNG and STARLUNG trials for stereotactic body radiotherapy (SBRT) of central and ultra-central lung tumours. Radiother. Oncol..

[25] Onishi H., Araki T., Shirato H., Nagata Y., Hiraoka M., Gomi K. (2004). Stereotactic hypofractionated high‐dose irradiation for stage I nonsmall cell lung carcinoma: clinical outcomes in 245 subjects in a Japanese multiinstitutional study. Cancer.

[26] Chang J.Y., Balter P.A., Dong L., Yang Q., Liao Z., Jeter M. (2008). Stereotactic body radiation therapy in centrally and superiorly located stage I or isolated recurrent non-small-cell lung cancer. Int. J. Radiat. Oncol. Biol. Phys..

[27] Lindberg K., Grozman V., Karlsson K., Lindberg S., Lax I., Wersäll P. (2021). The HILUS-trial—a prospective nordic multicenter phase 2 study of ultracentral lung tumors treated with stereotactic body radiotherapy. J. Thorac. Oncol..

[28] Giuliani M.E., Filion E., Faria S., Kundapur V., Toni Vu T.T.T., Lok B.H. (2024). Stereotactic radiation for ultra-central non-small cell lung cancer: a safety and efficacy trial (SUNSET). Int. J. Radiat. Oncol. Biol. Phys..

[29] Bezjak A., Paulus R., Gaspar L.E., Timmerman R.D., Straube W.L., Ryan W.F. (2019). Safety and Efficacy of a Five-Fraction Stereotactic Body Radiotherapy Schedule for Centrally Located Non-Small-Cell Lung Cancer: NRG Oncology/RTOG 0813 Trial. J. Clin. Oncol..

[30] Tekatli H., Haasbeek N., Dahele M., De Haan P., Verbakel W., Bongers E. (2016). Outcomes of hypofractionated high-dose radiotherapy in poor-risk patients with “ultracentral” non–small cell lung cancer. J. Thorac. Oncol..

[31] Lodeweges J.E., van Rossum P.S., Bartels M.M.J., van Lindert A.S., Pomp J., Peters M. (2021). Ultra-central lung tumors: safety and efficacy of protracted stereotactic body radiotherapy. Acta Oncol..

[32] Ladbury C., Sidiqi B., Cantrell N., Jones G., Skalina K.A., Fekrmandi F. (2025). Stereotactic body radiation therapy for primary lung cancer and metastases: a case-based discussion on challenging cases. Pract. Radiat. Oncol..

[33] Yan M., Louie A.V., Kotecha R., Ashfaq Ahmed M., Zhang Z., Guckenberger M. (2023). Stereotactic body radiotherapy for Ultra-Central lung Tumors: A systematic review and Meta-Analysis and International Stereotactic Radiosurgery Society practice guidelines. Lung Cancer.

[34] Regnery S., Ristau J., Weykamp F., Hoegen P., Sprengel S.D., Paul K.M. (2022). Magnetic resonance guided adaptive stereotactic body radiotherapy for lung tumors in ultracentral location: the MAGELLAN trial (ARO 2021-3). Radiat. Oncol..

[35] Lee G., Han Z., Huynh E., Tjong M.C., Cagney D.N., Huynh M.A. (2024). Widening the therapeutic window for central and ultra-central thoracic oligometastatic disease with stereotactic MR-guided adaptive radiation therapy (SMART). Radiother. Oncol..

[36] Breen W.G., Jeans E.B., Gergelis K.R., Garces Y.I., Park S.S., Merrell K.W. (2021). Ablative radiotherapy for ultracentral lung cancers: Dosimetric, geometric, and volumetric predictors of outcomes and toxicity. Radiother. Oncol..

[37] Cereno R.E., Mou B., Baker S., Chng N., Arbour G., Bergman A. (2023). Should organs at risk (OARs) be prioritized over target volume coverage in stereotactic ablative radiotherapy (SABR) for oligometastases? a secondary analysis of the population-based phase II SABR-5 trial. Radiother. Oncol..

