# Supplementary material for: Impact of tumour proximity to organs-at-risk in adaptive MR-guided SBRT for central lung tumours and metastases
Source: Clin Transl Radiat Oncol. 2025 Nov 15;57:101079. doi: 10.1016/j.ctro.2025.101079 (PMC12794038; doi:10.1016/j.ctro.2025.101079)
Supplement: Supplementary Data 1 [file mmc1.docx]

**Supplementary** **Table 1: Tumour and Treatment Planning Details per Patient**

|  | Central Tumour Location (Relative to OAR) | OAR in Contact with GTV | GTV Volume | GTV to PTV margin | OAR Overlapping with PTV | Planning Risk Volume (PRV) | PRV margin | No. of Fx | Dose prescription |
| --- | --- | --- | --- | --- | --- | --- | --- | --- | --- |
| 1 | PBT | PBT | 4.33 cc | 5 mm | PBT | PBT | 0 mm | 10 | 5 Gy to 50 Gy at 80% IDL |
| 2 | PBT | PBT, Oesophagus, Great vessels, Heart | 147.40 cc | 5 mm | PBT, Oesophagus, Great vessels, Heart | PBT, Oesophagus, Great vessels, Heart | 3 mm | 10 | 5 Gy to 50 Gy at 80% IDL |
| 3 | PBT | - | 31.24 cc | 5 mm | PBT (contact without overlap) | PBT | 0 mm | 10 | 5 Gy to 50 Gy at 80% IDL |
| 4 | PBT | - | 5.37 cc and 4.99 cc (2 GTVs) | 5 mm | Heart, PBT | Heart, PBT | 3 mm | 10 | 5 Gy to 50 Gy at 80% IDL |
| 5 | PBT | PBT | 1.17 cc | 5 mm | PBT, Great vessels | PBT | 0 mm | 10 | 5 Gy to 50 Gy at 80% IDL |
| 6 | PBT | PBT, Oesophagus, Great vessels | 11.02 cc | 3 mm | Oesophagus, PBT, Great vessels | Oesophagus, PBT | 3 mm | 10 | 5 Gy to 50 Gy at 80% IDL |
| 7 | PBT | PBT, Great vessels | 8.55 cc and 3.15 cc (2 GTVs) | 5 mm | PBT, Great vessels | PBT | 3 mm | 10 | 5 Gy to 50 Gy at 80% IDL |
| 8 | PBT | - | 12.35 cc | 5 mm | PBT | PBT | 3 mm | 10 | 5 Gy to 50 Gy at 80% IDL |
| 9 | PBT | - | 4.47 cc | 3 mm | - | - | - | 8 | 7.5 Gy to 60 Gy at 80% IDL |
| 10 | PBT | PBT | 22.46 cc and 32.02 cc (2 GTVs) | 5 mm | PBT | PBT | 3 mm | 10 | 5 Gy to 50 Gy at 80% IDL |
| 11 | PBT | PBT | 5.96 cc | 5 mm | PBT | PBT | 3 mm | 10 | 5 Gy to 50 Gy at 80% IDL |
| 12 | PBT | - | 2.93 cc | 5 mm | Heart | Heart | 0 mm | 10 | 5 Gy to 50 Gy at 80% IDL |
| 13 | PBT | - | 5.49 cc | 5 mm | - | - | - | 8 | 7.5 Gy to 60 Gy at 80% IDL |
| 14 | Trachea | - | 1.62 cc | 5 mm | Trachea, Oesophagus | Trachea, Oesophagus | 3 mm | 10 | 5 Gy to 50 Gy at 80% IDL |
| 15 | Trachea | Trachea, PBT, Great vessels | 3.45 cc | 5 mm | Trachea, PBT, Great vessels | Trachea, PBT | 3 mm | 10 | 5 Gy to 50 Gy at 80% IDL |
| 16 | Heart | - | 7.74 cc | 5 mm | - | - | - | 5 | 10 Gy to 50 Gy at 80% IDL |
| 17 | Heart | - | 2.31 cc | 5 mm | - | - | - | 3 | 15 Gy to 45 Gy at 65% IDL |
| 18 | Heart | - | 1.04 cc | 5 mm | - | Heart | 0 mm | 3 | 13.5 Gy to 40.5 Gy at 65% IDL |
| 19 | Heart | - | 1.13 cc | 5 mm | - | - | - | 3 | 15 Gy to 45 Gy at 65% IDL |
| 20 | Heart | - | 2.58 cc | 5 mm | Heart (contact without overlap) | Heart | 0 mm | 5 | 10 Gy to 50 Gy at 80% IDL |
| 21 | Heart | Heart | 134.83 cc | 3 mm | Heart | Heart | 3 mm | 10 | 5 Gy to 50 Gy at 80% IDL |
| 22 | Heart | - | 0.97 cc | 5 mm | Heart | - | - | 5 | 10 Gy to 50 Gy at 80% IDL |
| 23 | Heart | Heart, Great vessels | 14.81 cc | 5 mm | Heart, Great vessels | Heart, Great vessels | 0 mm | 10 | 5 Gy to 50 Gy at 80% IDL |
| 24 | Heart | Heart | 23.68 cc | 5 mm | Heart | Heart | 2 mm | 10 | 5 Gy to 50 Gy at 80% IDL |
| 25 | Heart | Heart | 5.09 cc | 5 mm | Heart | Heart | 2 mm | 10 | 5 Gy to 50 Gy at 80% IDL |
| 26 | Heart | - | 2.59 cc | 5 mm | Heart | Heart | 0 mm | 10 | 5 Gy to 50 Gy at 80% IDL |
| 27 | Great vessels | - | 5.71 cc | 5 mm | Great vessels (contact without overlap) | - | - | 5 | 10 Gy to 50 Gy at 80% IDL |
| 28 | Great vessels | - | 2.22 cc | 3 mm | - | - | - | 5 | 10 Gy to 50 Gy at 80% IDL |
| 29 | Great vessels | - | 22.82 cc | 5 mm | - | - | - | 8 | 7.5 Gy to 60 Gy at 80% IDL |
| 30 | Great vessels | - | 2.79 cc | 5 mm | Great vessels | Great vessels | 0 mm | 5 | 10 Gy to 50 Gy at 80% IDL |
| 31 | Great vessels | Great vessels | 27.52 cc | 5 mm | Great vessels | Great vessels | 0 mm | 10 | 5 Gy to 50 Gy at 80% IDL |
| 32 | Great vessels | - | 12.89 cc | 5 mm | Great vessels | Great vessels | 0 mm | 10 | 10 Gy to 50 Gy at 80% IDL |
| 33 | Brachial plexus (BP) | - | 3.29 cc | 3 mm | - | BP | - | 5 | 10 Gy to 50 Gy at 80% IDL |
| 34 | BP | BP | 10.86 cc | 5 mm | BP | BP | 3 mm | 10 | 5 Gy to 50 Gy at 80% IDL |
| 35 | BP | BP | 1.24 cc | 5 mm | - | BP | 3 mm | 10 | 5 Gy to 50 Gy at 80% IDL |
| 36 | BP | - | 3.03 cc | 5 mm | BP | BP | 3 mm | 10 | 5 Gy to 50 Gy at 8% IDL |

BP - Brachial plexus; Fx - fractions; GTV - Gross Tumour Volume; IDL - Isodose line; PTV - Planning Target Volume; OAR - Organ-at-Risk, PRV - Planning Risk Volume, Gy - Gray, PBT - Proximal Bronchial Tree
